# Why I teach the controversy: using creationism to teach critical thinking

**DOI:** 10.3389/fpsyg.2015.00793

**Published:** 2015-06-17

**Authors:** P. Lynne Honey

**Affiliations:** Department of Psychology, MacEwan University, Edmonton, AB, Canada

**Keywords:** teaching, psychology, creationism, critical thinking, evolution, science education

## Abstract

Creationism and intelligent design are terms used to describe supernatural explanations for the origin of life, and the diversity of species on this planet. Many scientists have argued that the science classroom is no place for discussion of creationism. When I began teaching I did not teach creationism, as I focused instead on my areas of expertise. Over time it became clear that students had questions about creationism, and did not understand the difference between a scientific approach to knowledge and non-scientific approaches. This led me to wonder whether ignoring supernatural views allowed them to remain as viable “alternatives” to scientific hypotheses, in the minds of students. Also, a psychology class is an ideal place to discuss not only the scientific method but also the cognitive errors associated with non-science views. I began to explain creationism in my classes, and to model the scientific thought process that leads to a rejection of creationism. My approach is consistent with research that demonstrates that teaching content alone is insufficient for students to develop critical thinking and my admittedly anecdotal experience leads me to conclude that “teaching the controversy” has benefits for science students.

When I began teaching, I was eager to share my excitement about psychology and train students in scientific methods. That excitement and eagerness has not changed. Other things have changed, however. I have learned to love technology that allows me to teach and communicate with students in different ways. I have tried various methods, and continue to revise my materials. I’ve adopted new approaches, and abandoned others. I have also had a significant change in my attitude toward the discussion of creationism in my classroom, and that change is the focus of this article.

I will use the term creationism to refer mainly to typical Judeo-Christian accounts of the origin of life, found in the biblical book of Genesis. While there are other creation stories in various cultures and religions, this version of creationism is most associated with political movements intended to suppress the teaching of evolution or to include the teaching of supernatural accounts as alternatives to evolution (Ruse, 2014). I also include in my definition of creationism the concept of intelligent design. Although intelligent design does not name a specific entity as the originator of life, it does propose that life was initiated by a “master intellect” that operates outside of known natural laws. While this belief system is not associated with a specific religion, in practice most proponents of intelligent design are Christians who use the term intelligent design to mask the religious nature of their arguments against evolution (Numbers, 2006).

I won’t discuss the validity of evolutionary theory or creationist beliefs. That matter is settled, from a scientific perspective if not from the perspective of popular opinion (see Miller, 2008). There is no scientific evidence to support any creationist theories, whereas “Nothing in biology makes sense except in the light of evolution” (Dobzhansky, 1973, p. 125). What I will discuss is whether creationist ideas should be discussed in science classrooms. I will present positions from scientists, educators, and creationists, and I will discuss my use of creationism in the classroom in the context of teaching students about evolution and psychology.

## Teaching the Controversy

The slogan “Teach the Controversy” originated around the turn of the 21st century, and is reflected in a newspaper article by intelligent design advocate Meyer (2002) in which he states that educators should present arguments both for and against evolutionary theory, and that educators should be permitted to teach intelligent design as a competing theory. He argued that because there is a Supreme Court mandate to teach scientific critiques of prevailing theories, and that federal policy dictates that curriculum should help students understand controversies, that teachers should be expected to teach evolution as if there is an actual scientific controversy about evolutionary theory. As noted by Scott and Branch (2003), this proposal is consistent with ongoing lobbying by creationist groups in the U.S., and it is cloaked in a veil of fairness about presenting both sides of an argument. While scientists should teach scientific critiques of prevailing theories, there should be no obligation to teach non-scientific critiques as if they were equally valid.

Scientists have reacted strongly to suggestions that creationist views be taught alongside scientific theories. Dawkins and Coyne (2005) have stated emphatically that there is no place in a science classroom for creationism. If science instructors were to take the “10 min to exhaust the case for (Intelligent Design)” (Dawkins and Coyne, 2005, One side may be wrong, para. 20) then they lend legitimacy to creationism by its mere presence in the science classroom. This is consistent with Grayling’s (2014) position that broadening the conversation to include non-scientific approaches validates those non-scientific approaches and provides them with institutionalized importance. Scott (2007) warns teachers about the potential incursion of “Teach the Controversy” policies that may affect curriculum: Under the guise of recommendations to teach critical thinking, these proposals present the false view that there is any question about *whether* evolution occurs. She writes:

It might be a useful critical thinking exercise for students to debate actual scientific disputes about patterns and processes of evolution, as long as they have a solid grounding in the basic science required…It would, however, not be a good critical thinking exercise to teach students that scientists are debating whether evolution takes place: on the contrary, it would be gross miseducation to instruct students that the validity of one of the strongest scientific theories is being questioned. (Scott, 2007, pp. 313–314)

More recently, Coyne (2014) revealed his disappointment when Bill Nye (The Science Guy) debated young earth creationist Ken Ham in a widely publicized event (National Public Radio, 2014). Coyne argues, like Dawkins (2006), that by engaging in public debate, scientists lend credibility to creationists and elevate their arguments to a status that they don’t deserve. By acknowledging creationist arguments, and treating them as threats to scientific knowledge, we run the risk of validating them.

In short, rejection of the Teach the Controversy movement is based on two key principles. First, there is no controversy. Evolution is a robust and well-supported theory that has undergone rigorous testing, and is a unifying theory in science. Second, creationism does not belong in science curricula. Entertaining non-science notions is dangerous because discussing those notions in a science classroom risks legitimizing them. While I completely endorse the first principle, I have changed my mind about the second principle. This is not to say that I would ever support creationism as a valid scientific theory, but rather that there is value in discussing creationism in the science classroom.

Until 2005, I was inclined to teach evolution as if creationism did not exist. It was not part of my teaching materials, and if students asked questions about creationism, I tended to respond that those questions were not suitable for a science classroom. I did entertain those questions outside of class, (where I made it very clear that I was talking about my opinions, rather than course material), but I drew a hard line at the classroom door. In 2005, I noticed an increase in the number of questions that students raised about creationism, likely in response to the media attention that surrounded the trial in Dover, Pennsylvania where a parent took the Dover Board of Education to court over the Board’s decision to use a creationist textbook in science classes (see Miller, 2008).

It is worth noting that creationist views are not rare. Approximately one-third of American adults reject evolution and more than half do not completely endorse evolution (Miller et al., 2006). Although the rates of creationist belief are higher in the United States than most other countries, there is no consensus on evolution in Canada or Britain, either. According to a recent poll (Angus, 2012), 22% of Canadians and 17% of Britons endorse some version of creationism. Compared to the 51% of Americans who endorse creationism, such numbers are modest, but still concerning. There are regional differences too. In the United States, residents of the south are most likely to hold creationist views. In Britain, there is less variation but London has the highest proportion of creationists. In Canada, the province of Alberta—where I teach—has the lowest endorsement of evolution at 48% of the population, and 17% of the population is “not sure” (Angus, 2012). Thus, there is a reasonably large proportion of the population who are at least unsure about evolution.

After the Dover trial, I made a decision to use one lecture to explain to students why creationism is not science. I had mixed feelings about bringing non-science into the classroom, but after observing apparent benefits to student learning I have decided to continue. While I respect the views and admonitions of my colleagues who avoid discussing creationism, I think that inclusion of creationism is still consistent with Scott’s (2007) advice described above. Perhaps because I teach psychology, I have a different perspective on appropriate topics.

## Teaching Psychology

Psychology is a science that studies human and animal behavior, and the application of that science in clinical, educational, and other settings. I find that teaching psychology is very “meta” because the practice of teaching is informed by the very content that we aim to teach. Evidence from psychological studies is necessary to understand why there is value in not only delivering content, but also in fostering critical thinking skills in the classroom.

While my colleagues in biology might recoil at the notion of discussing non-scientific thought in their classrooms, the psychology class differs from other sciences in one very important way; the topic of our inquiry is behavior, including thoughts. Any psychologist who teaches cognition, or development, or social behavior is expected to cover content about cognitive biases and decision-making. There is a movement to promote critical thinking skills (Lilienfeld, 2012), for psychologists to work toward debiasing public thought (Lilienfeld et al., 2009) and debunking pseudoscience (Lilienfeld, 2005). While it is important to train our students by discussing controversies within our fields and between competing scientific theories (Scott and Branch, 2003; Dawkins and Coyne, 2005), it is also useful to train our students in detecting pseudoscience and to argue against it (Lilienfeld, 2012).

Among the various psychology courses that I have taught over the years, one of my favorites is a course on evolution and human behavior. Coming from an experimental background, trained in animal learning and behavior, I understand human behavior as an interaction between our evolved tendencies and our environments. There has been controversy about this field from the time that E.O. Wilson coined the term “sociobiology” (see Confer et al., 2010) and I have always enjoyed teaching *that* controversy as a way of demonstrating scientific thought and progress. For example, I have students generate hypotheses about sex differences, and we work through methods of testing those hypotheses to distinguish between valid tests of biological sex differences, and “just so stories” that lack evidence and ignore the role of learning. Within this course, I also have the opportunity to discuss cognitive biases as evolved heuristics (e.g., Cosmides, 1989) that result in predictable errors in judgment. In this context, where I model scientific logic and provide information about human errors in cognition, a discussion of creationism seems perfectly appropriate.

Creationism is presented as a sociopolitical controversy rather than a scientific controversy. I emphasize that there is no question about the validity of evolution as an explanatory model, and I present creationism as a political or “denialist” movement (Diethelm and McKee, 2009) rather than a competing theory with its own strengths and evidence. I then present several common assertions from creationism (e.g., that there are no transitional fossils), and refute them using scientific evidence. At the same time, I explain several of the common logical fallacies that are evident in creationist arguments. I encourage students to ask questions, and force me to defend my statements. I then ask them to attempt to generate hypotheses and tests of creationism. Their struggles with this task lead them, logically, to the conclusion that many creationist assertions are unfalsifiable and therefore non-scientific.

Although it feels ironic, the following anecdotal evidence illustrates my approach. I presented an *ad hoc* reasoning fallacy, in which some creationists have responded to fossil evidence with statements that those fossils must have been put there (by supernatural forces) to trick or test the faith of believers. After I pointed out that original statements are revised in order to preserve the key belief (creationism), a student indicated that he was confused. He thought that being able to revise your thinking was important in science, and he couldn’t understand why a creationist revising his thinking was bad, but a scientist revising her thinking was good. I could see several other students tilt their heads to ponder this point, and realized that I needed to back up and revisit the concept of falsifiability: using evidence to *reject* a hypothesis is scientific, whereas creating an explanation for evidence in order to *defend* the original hypothesis is not scientific. Scientists use *ad hoc* reasoning to refine and strengthen theory but not to insulate a weak theory from valid criticism. We then discussed several situations, including decisions about medical treatments or support for political parties, in which people should reject their hypotheses based on available evidence, but instead create additional explanatory layers in order to avoid changing their minds. Students have often told me that they knew that creationist arguments were flawed, but they didn’t know how to articulate their discomfort. Students have expressed appreciation for being given the tools to better argue their positions.

There are several reasons why this approach is valuable. It is consistent with the “teaching as persuasion” model (Alexander et al., 2002) which allows multiple views to be discussed, and then some are rejected for lack of substantiation. It also debunks pseudoscientific beliefs (Lilienfeld, 2005), by providing direct evidence against them. Further, by practicing arguments against standard creationist assertions, students benefit from the inoculation effect (see Jost and Hardin, 2011) and should therefore be better able to refute similar arguments in the future.

I think that all science educators could benefit from strategies associated with teaching critical thinking. Teaching content alone does not teach students to think like scientists. Development of critical thinking requires that students learn content along with an opportunity to practice the metacognitive strategies associated with critical thinking (Willingham, 2007). Dawkins and Coyne (2005) argued that creationism “no more belongs in a biology class than alchemy belongs in a chemistry class” (para. 6). I argue that alchemy could belong in chemistry classrooms, if it demonstrated why some methods of gathering knowledge are more valid than others. Again, my psychology training may bias my views.

Psychology has often been considered a “soft” science, contra-sted with the “hard” sciences of chemistry and physics, and is often snubbed from the life sciences of biology and medicine (Lilienfeld, 2012). Perhaps as a consequence of an inferiority complex, academic psychology has focused on training students in research methods to a much greater extent than other sciences (Winston and Blais, 1996). The table of contents for most psychology textbooks will reveal a chapter on methodology, and the history of the discipline. There are many famous examples in psychology of rejected hypotheses and paradigm shifts. The practice of phrenology—a method for inferring personality and behavior based on the shape of one’s skull—was once rather common and popular (see Goodwin, 2005). It was soundly rejected because of evidence presented by physiologists and others. This rejection provides important lessons about differences between scientific approaches to psychology and non-scientific approaches. Our understanding of psychology is based not only on verifiable facts, but also on rejection of previous ideas. Just as phrenology belongs in the psychology classroom, so might creationism.

## Creationism, Critical Thinking, and the Language of Science

The argument for teaching the controversy implies that science is a matter of opinion debated by opposing parties with equally valid positions. When creationists describe intelligent design as a theory equal to that of evolutionary theory, they demonstrate their ignorance of the language of science even as they use its words (Barnes, 2014). By using jargon inappropriately, they deceive people who assume that everyone uses those words consistently. When I talk about the theory of evolution, I refer to an overarching set of principles and predictions that allows me to understand the natural world. A scientific theory is not a tentative statement, but rather is supported by empirical evidence. When I talk about hypotheses I refer to predictions, based on theory, that require testing. The hypothesis is tentative, and may be refuted by evidence, but the failure of a hypothesis does not necessarily degrade a theory. When creationists refer to evolution as “just a theory” they ignore stacks of evidence that have supported that theory since Darwin first described it. A non-scientist who hears a creationist and a scientist each use “theory” to refer to their positions can be understandably confused.

Positioning intelligent design as scientific theory is inappropriate, because it lacks empirical support and portions of it are untestable. It is critically important that our students be given the opportunity to understand the definitions of words like “theory,” “hypothesis,” “proof,” and “evidence” and to be able to detect when those words are being used inaccurately. Learning to discriminate between appropriate and inappropriate uses of those words requires more than simply memorizing definitions. If students apply that knowledge and practice the skills associated with science, they will generate a deeper understanding. If I simply state that creationism is not scientific, then I ask my students to take my word for it because I am the authority as a scientist. They may adopt my views, because I’m an authority, but not because they have internalized the logic that led to my views (McCaffree and Saide, 2014). When I allow them to apply the scientific method to creationism, they practice being scientists themselves. Although critical thinking is very difficult to teach, and often does not transfer across domains (Halpern, 1998), there is incremental value in providing multiple opportunities to practice critical thinking while learning new scientific content (Willingham, 2007).

My position is not restricted to the use of creationism to teach critical thinking. The same position could apply to other anti-science views including those opposed to vaccines or other validated medical procedures, to climate change denialism, or to other supernatural explanations for natural phenomena. As educators, we can take the opportunity to tackle topics that students may see in the media, on social media, or around the dinner table, and model our thought processes as we explain how scientists come to conclusions. We can emphasize that not all statements are equally valid, not all “authorities” are equally authoritative, and not all hypotheses are equally testable. We can also allow students to practice their logic skills, and apply them to new topics that arise with each poorly informed Facebook meme, or celebrity fad. Non-science and anti-science views do have a place in the science classroom, because they can be used to train students in the logic associated with scientific thought.

### Conflict of Interest Statement

The author declares that the research was conducted in the absence of any commercial or financial relationships that could be construed as a potential conflict of interest.

## References

[B1] Alexander P. A. Fives H. Buehl M. M. Mulhern J. (2002). Teaching as persuasion. Teach. Teach. Educ. 18, 795–813. 10.1016/S0742-051X(02)00044-6

[B2] Angus R. (2012). Britons and Canadians More Likely to Endorse Evolution than Americans. Available at: http://angusreidglobal.com/wp-content/uploads/2012/09/2012.09.05_CreEvo.pdf [accessed September 5, 2012].

[B3] Barnes R. M. (2014). Do unique definitions of ‘science’ and ‘proof’ allow creationists to disregard evidence of evolution? Skeptic 19, 49–53.

[B4] Confer J. C. Easton J. A. Fleischman D. S. Goetz C. D. Lewis D. M. Perilloux C. (2010). Evolutionary psychology. Controversies, questions, prospects, and limitations. Am. Psychol. 2, 110–126. 10.1037/a0018413 20141266

[B5] Cosmides L. (1989). The logic of social exchange: Has natural selection shaped how humans reason? Studies with the Wason selection task. Cognition 31, 187–276. 2743748 10.1016/0010-0277(89)90023-1

[B6] Coyne J. (2014). Bill Nye Talks about His Upcoming Debate with Ken Ham (weblog). Available at: https://whyevolutionistrue.wordpress.com/2014/01/08/bill-nye-talks-about-his-upcoming-debate-with-ken-ham/ (accessed January10, 2015)

[B7] Dawkins R. (2006). Why I Won’t Debate Creationists (weblog). Available at: http://old.richarddawkins.net/articles/119-why-i-won-39-t-debate-creationists (accessed January 10, 2015)

[B8] Dawkins R. Coyne J. (2005). One Side Can be Wrong. The Guardian (online edition). Available at: http://www.theguardian.com/science/2005/sep/01/schools.research (accessed September 1, 2005).

[B9] Diethelm P. McKee M. (2009). Denialism: what is it and how should scientists respond? Eur. J. Pub. Health 19, 2–4. 10.1093/eurpub/ckn139 19158101

[B10] Dobzhansky T. (1973). Nothing in biology makes sense except in the light of evolution. Am. Biol. Teach. 75, 87–91. 10.2307/4444260

[B11] Goodwin C. J. (2005). A History of Modern Psychology, 2nd Edn. New Jersey, NJ: Wiley.

[B12] Grayling A. C. (2014). Teach the Controversy (video podcast). Available at: https://richarddawkins.net/2014/06/a-c-grayling-teach-the-controversy-4/ [accessed June 5, 2014].

[B13] Halpern D. (1998). Teaching critical thinking for transfer across domains: disposition, skils, structure training, and metacognitive monitoring. Am. Psychol. 53, 449–455. 10.1037/0003-066X.53.4.449 9572008

[B14] Jost J. T. Hardin C. D. (2011). On the structure and dynamics of human thought: the legacy of William J. McGuire for social and political psychology. Polit. Psychol. 32, 21–57. 10.1111/j.1467-9221.2010.00794.x

[B15] Lilienfeld S. O. (2005). The 10 commandments of helping students distinguish science from pseudoscience in psychology. APS Obs. 18, 39–40; 49–51.

[B16] Lilienfeld S. O. (2012). Public skepticism of psychology: why many people perceive the study of human behavior as unscientific. Am. Psychol. 67, 111–129. 10.1037/a0023963 21668088

[B17] Lilienfeld S. O. Ammirati R. Landfield K. (2009). Giving debiasing away: can psychological research on correcting cognitive errors promote human welfare? Perspect. Psychol. Sci. 4, 390–398. 10.1111/j.1745-6924.2009.01144.x 26158987

[B18] McCaffree K. Saide A. (2014). Why is critical thinking so hard to teach? Skeptic 19, 54–57.

[B19] Meyer S. C. (2002). Teach the controversy. Cincinnati Enq, 30, D3.

[B20] Miller K. R. (2008). Only a Theory: Evolution and the Battle for America’s Soul. New York, NY: Penguin.

[B21] Miller J. D. Scott E. C. Okamoto S. (2006). Public acceptance of evolution. Science 313, 765–766. 10.1126/science.1126746 16902112

[B22] National Public Radio (2014). Watch the Creationism vs. Evolution Debate: Ken Ham and Bill Nye (blog post with embedded video). Available at: http://www.npr.org/blogs/thetwo-way/2014/02/04/271648691/watch-the-creationism-vs-evolution-debate-bill-nye-and-ken-ham (accessed December 12, 2015)

[B23] Numbers R. L. (2006). The Creationists: From Scientific Creationism to Intelligent Design. Cambridge, MA: Harvard University Press.

[B24] Ruse M. (2014). “Creationism,” in The Stanford Encyclopedia of Philosophy, ed. Zalta E. N. Available at http://plato.stanford.edu/archives/sum2014/entries/creationism/ (accessed February 12, 2015)

[B25] Scott E. C. (2007). What’s wrong with the “Teach the Controversy” slogan? McGill J. Educ. 42, 307–315.

[B26] Scott E. C. Branch G. (2003). Evolution: what’s wrong with ‘teaching the con-troversy’. Trends Ecol. Evol. 18, 499–502. 10.1016/S0169-5347(03)00218-0

[B27] Willingham D. T. (2007). Critical thinking: why is it so hard to teach? Am. Educ. Summer 8–19.

[B28] Winston A. S. Blais D. J. (1996). What counts as an experiment? A transdisciplinary analysis of textbooks, 1930–1970. Am. J. Psychol. 109, 599–616. 10.2307/1423397

